# Studying the viability and growth kinetics of vancomycin-resistant *Enterococcus faecalis* V583 following femtosecond laser irradiation (420–465 nm)

**DOI:** 10.1007/s10103-024-04080-5

**Published:** 2024-05-29

**Authors:** Ahmed O. El-Gendy, Sarah Ezzat, Fatma Abdel Samad, Ola Ali Dabbous, Jonathan Dahm, Michael R. Hamblin, Tarek Mohamed

**Affiliations:** 1 Laser Institute for Research and Applications LIRA, Beni-Suef University, Beni-Suef, 62511 Egypt; 2 Department of Microbiology and Immunology, Faculty of Pharmacy, Beni-Suef University, Beni-Suef, 62514 Egypt; 3 Department of Medical Applications of Lasers, National Institute of Laser Enhanced Science (NILES), Cairo University, Giza, 12611 Egypt; 4 Lightstream Photonics, Boulder, CO, CO 80302 USA; 5 Laser Research Centre, Faculty of Health Science, University of Johannesburg, Doornfontein, 2028 South Africa

**Keywords:** Femtosecond Laser light, Antibacterial photoinactivation, Viable bacterial counts, Bacterial Growth Kinetics, Antibiotic-resistance, *Enterococcus faecalis*

## Abstract

*Enterococcus faecalis* is among the most resistant bacteria found in infected root canals. The demand for cutting-edge disinfection methods has rekindled research on photoinactivation with visible light. This study investigated the bactericidal activity of femtosecond laser irradiation against vancomycin-resistant *Enterococcus faecalis* V583 (VRE). The effect of parameters such as wavelength and energy density on the viability and growth kinetics of VRE was studied to design an optimized laser-based antimicrobial photoinactivation approach without any prior addition of exogenous photosensitizers. The most effective wavelengths were 430 nm and 435 nm at a fluence of 1000 J/cm^2^, causing a nearly 2-log reduction (98.6% and 98.3% inhibition, respectively) in viable bacterial counts. The colony-forming units and growth rate of the laser-treated cultures were progressively decreased as energy density or light dose increased at 445 nm but reached a limit at 1250 J/cm^2^. At a higher fluence of 2000 J/cm^2^, the efficacy was reduced due to a photobleaching phenomenon. Our results highlight the importance of optimizing laser exposure parameters, such as wavelength and fluence, in bacterial photoinactivation experiments. To our knowledge, this is the first study to report an optimized wavelength for the inactivation of VRE using visible femtosecond laser light.

**Supplementary Information:**

The online version contains supplementary material available at 10.1007/s10103-024-04080-5.

## Introduction

The bacterium *Enterococcus faecalis* is widespread throughout the gastrointestinal tract, including the oral cavity [1]. It has been reported that *E. faecalis* is the most common microorganism found in infected root canals and in recurrent cases of apical periodontitis, with a prevalence ranging from 24–77% [2]. This is because *E. faecalis* can invade deeply into dentinal tubules and can resist intracanal disinfection procedures during routine endodontic treatment [3]. *E. faecalis* can survive in filled root canals without the support of any other bacteria [4]. Furthermore, *E. faecalis* is well-known for its resilience in the environment and can occur naturally in water sources such as rivers, lakes, and groundwater [5].

To prevent *E. faecalis* survival in dental settings and drinking water systems, proper disinfection control measures should be followed. In dentistry, irrigation with sodium hypochlorite, sterilization of dental instruments, using disposables when necessary, and adhering to stringent hand hygiene guidelines should all be standard practice. Sodium hypochlorite is used as the main irrigant during root canal therapy. However, it can be hazardous to the host cells of the periapical region if they come into contact [6, 7].

Vancomycin-resistant Enterococci (VRE) such as *E. faecalis* V583 are challenging to treat and can cause life-threatening infections, especially in immunocompromised individuals. Recently the spread of multidrug-resistant microorganisms in healthcare settings has become a serious threat to public health, so it is now urgent that new anti-infective strategies be discovered. Moreover, new methods of treating endodontic infections in periapical tissues are also needed [8].

In the search for new antibacterial technologies, light-based techniques have been the subject of much study [9]. Optical irradiation using visible-light wavelengths has been widely proposed as an antibacterial technique [10, 11]. Endoscopically administered light therapy has previously been used to treat gastric *Helicobacter pylori* infection [12], in the treatment of urinary tract infection in a rat model [13], and in controlling bacterial biofilm growth on catheter material [14].

One promising light candidate is femtosecond laser-based antimicrobial photoinactivation (aPI) [15–19], which is actively being investigated for the treatment of resistant microbes [20–22]. aPI is a widely accepted, simple, non-invasive, and inexpensive treatment method that causes minimal to no harm to host tissue [23]. Additionally, microbes are unlikely to develop resistance to aPI [24–26]. The term "laser-based aPI" refers to a process whereby reactive oxygen species (ROS) are produced without the addition of any exogenous photosensitizers (PS), but instead relies on naturally occurring PS within the bacteria. Mechanistically it is similar to antimicrobial photodynamic therapy (aPDT), which does require the addition of PS. Both approaches can be used to kill specific pathogens, using ROS produced through two simultaneously competing photochemical processes [27]. Type I involves electron transfer reactions producing reactive free radicals, while Type II involves an energy transfer reaction producing singlet oxygen (_1_O^2^) [28]. In both these procedures, light energy is transformed into chemical energy via the interaction of laser radiation with absorbing compounds [15]. The release of ROS inside the bacterial cells causes oxidative stress, which ultimately results in cell death [29].

These photochemical processes require an energy-absorbing platform known as a photosensitizer (PS). The correct wavelength and intensity of light can trigger photochemical and photophysical events inside the microbial cell by activating these PS molecules [30, 31]. As the technology behind pulsed laser systems evolves, it is possible to control more and more laser characteristics, including wavelength, power density, pulse duration, exposure time, energy density, and laser polarization, for specific applications. The major goal of all researchers in this field is to identify the optimal light wavelength, dose and fluence, for preventing bacterial growth and multiplication while minimizing adverse effects to host tissue.

It was previously believed that radiation in the 405–410 nm range was the most effective visible spectral region for the photoinactivation of microbes [32–39]. However, recent research has suggested that the 420–470 nm spectral range can also kill bacteria without the use of exogenous PS [40–42]. Nevertheless, the visible spectrum from 420 to 465 nm has not been thoroughly investigated.

Moreover, it's essential to highlight the unique advantages of femtosecond laser technology over LED light. Femtosecond lasers offer precise control over pulse duration, enabling targeted and efficient delivery of light energy to bacterial cells. This precise control is crucial for maximizing ROS generation while minimizing damage to surrounding host tissue. Furthermore, femtosecond lasers provide superior spatial and temporal resolution compared to LEDs, allowing for highly localized and rapid bacterial photoinactivation. This capability is particularly advantageous in clinical settings where precise targeting of microbial pathogens is essential for successful treatment outcomes. While LEDs may offer affordability and versatility, their broader emission spectra and less precise pulse control may limit their effectiveness for certain aPI applications. In contrast, femtosecond lasers provide a sophisticated platform for fine-tuning treatment parameters and optimizing bacterial photoinactivation [17, 18, 22].

To date, very few reports have identified the optimum femtosecond laser parameters that could kill bacterial pathogens, particularly VRE. Most reports have focused on targeting bacterial porphyrins with light in the range of 390–420 nm, which is not the case when dealing with *E. faecalis* which does not contain any free porphyrins*,* but where flavins are the main absorbing molecules that could be targeted [43].

Therefore, this work set out to examine to what extent femtosecond laser irradiation could eradicate VRE. To determine the optimal laser-based bactericidal parameters, the effect of varying wavelength and energy density on the growth kinetics and viability of VRE was examined in bacterial suspensions in vitro.

## Materials and Methods

### Microorganisms and culture conditions

In the present study, the infectious bacterial species, vancomycin-resistant *Enterococcus faecalis* V583 (ATCC 700802), was cultured in Brain Heart Infusion broth and incubated at 37° C for 18 h. 100 µL of the overnight bacterial suspension was diluted in 10 mL 0.9% NaCl to bring the turbidity down to 0.5 McFarland units. Laser irradiation was performed by transferring 200 µL aliquots of the diluted suspension, using sterile pipette tips, into individual wells of a 96 microtiter plate.

## Preparation and installation of a laser system

Laser pulses were generated using a Spectra-Physics INSPIRE HF100 laser system, which was pumped by a mode-locked femtosecond Ti: sapphire MAI TAI HP laser, with 1.5–2.9 W average power, 80 MHz repetition rate, and wavelength range 690 to 1040 nm.

**Fig. 1:**
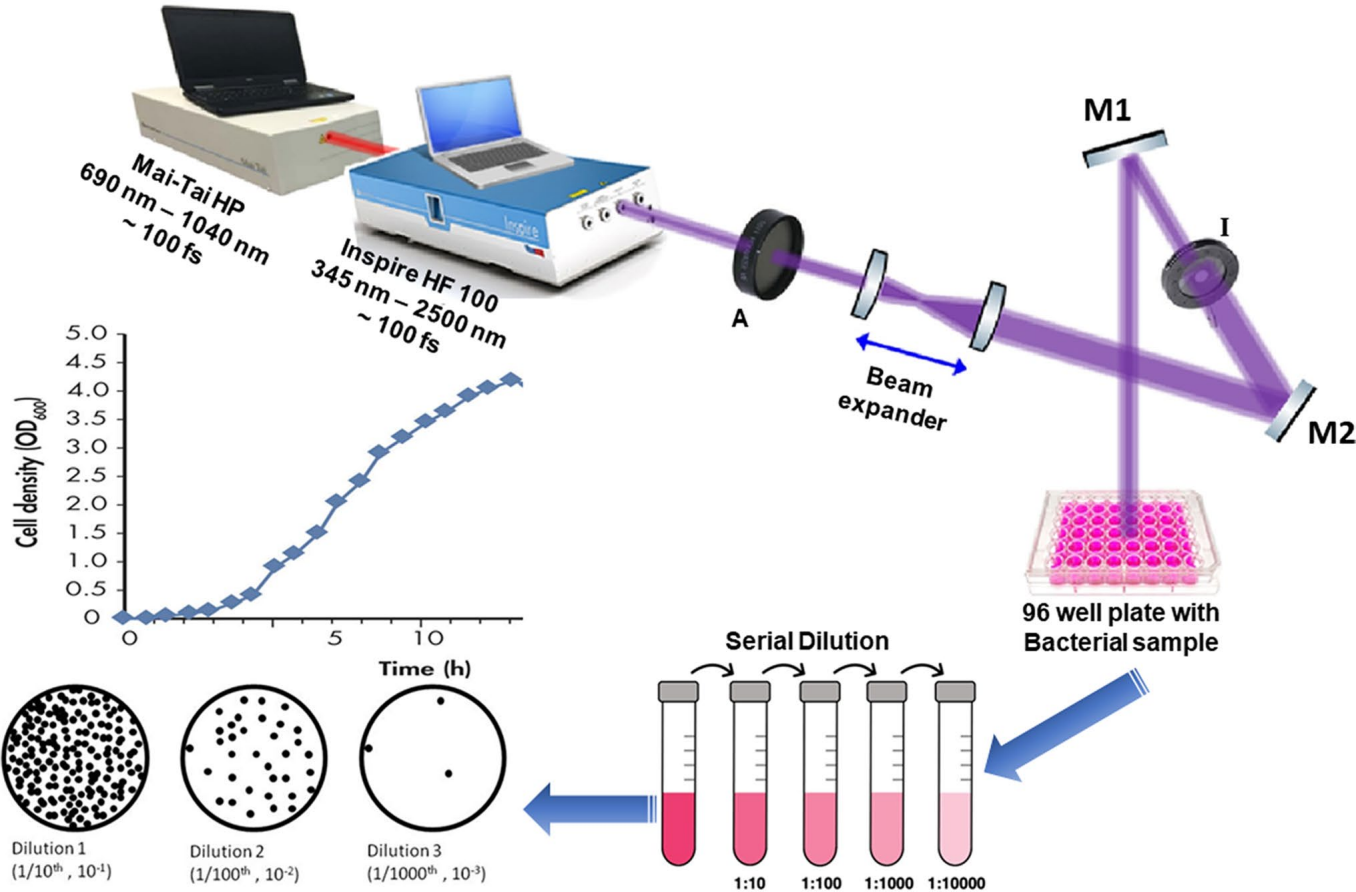
The experimental setup is depicted schematically. Components include an attenuator (A), highly reflective mirrors (M1 and M2), and an iris (I)

To achieve uniform interaction with the laser light, the bacterial suspensions were irradiated by positioning the laser beam about 10 cm above each well of a 96 well plate, as illustrated in Fig. 1.

A beam expander composed of two convergent lenses was used to increase the diameter of the original laser beam from 2 to 20 mm. An adjustable iris **I** was used to set the laser beam diameter at 6 mm, to match the diameter of each well of the microtiter plate, and a laser beam attenuator **A** was used to regulate the laser intensity delivered to the samples. As shown in Fig. 1, the laser beam was directed at the samples with the help of highly reflective mirrors **M1** and **M2**. A Newport 843R power meter was used to determine the precise output of the laser.

### The effect of varying laser irradiation parameters on *E. faecalis*

Horizontally polarised Gaussian laser pulses of 100 fs pulse duration and 80 MHz repetition rate were used to irradiate *E. faecalis* inoculated 96 well plates at a variety of wavelengths. A comprehensive analysis was conducted, comparing the viable bacterial counts, growth curves, and growth rate analysis of *E. faecalis* irradiated with 420, 425, 430, 440, 445, 452, 455, 460 and 465 nm, each at a constant energy density of 1000 J/cm^2^, to determine the most effective wavelengths. For the wavelengths of 420, 425, 430, 435, 440, and 445 nm, a higher energy density of 2000 J/cm^2^ was also explored. The effect of increasing energy density to potentiate the efficacy of the wavelength 445 nm was investigated using energy densities of 100, 250, 750, 1000, 1250, and 2000 J/ cm^2^.

### Determining and analyzing growth kinetics

The plates were incubated at 37° C for 16 h after each treatment. Every 30 min during this incubation, the optical density of all the samples was measured at 620 nm using an FC Microplate reader (Thermo Fisher Scientific SN: 357–906,489). To track the development and proliferation of the bacteria over time, growth curves and maximum growth kinetics at specific time points (μ_max_) were plotted. Each experiment was conducted in triplicate under a laminar flow hood (Class II biological safety cabinet, MSC-Advantage). The following equation was used to obtain μ_max_:

$${{\text{X}}}_{{\text{t}}} = {{\text{X}}}_{0} exp.({\upmu }_{{\text{max}}}.{\text{t}})$$

(X_t_ is the growth absorbance at a specified time point; X_0_ is the initial growth absorbance; t is the time at which μ_max_ obtained).

### Viable bacterial counts and assessment of photoinactivation effect

To measure the number of viable bacteria, 100 μL of each control and femtosecond laser irradiated sample were serial tenfold diluted using tubes containing 900 μL of sterile 0.9% NaCl (up to 1:10,000,000 dilutions). Then, 0.1 mL of each dilution was immediately plated on nutrient agar. After incubating the plates at 37 °C in an aerobic environment for 24 h, the total number of bacteria was counted. Colony-forming unit (CFU) counts were used to determine bacterial viability in test and control samples, with results given as log CFU/mL or % CFU/mL compared to the control. All experiments were carried out in triplicate.

### Statistical analysis and graphical representation

GraphPad Prism 7 was used for a one-way analysis of variance (ANOVA) test, followed by a Tukey test (*P* < 0.05 were considered significant), to compare the means of the treatment groups to those of the control group. Three independent runs of each experiment were conducted. Data visualization was plotted using GraphPad Prism 7 software.

## Results and Discussion

*E. faecalis*, a type of Gram-positive bacteria commonly found in dentistry, is highly resilient, and is blamed for the failure of 67% of cases of root canal treatment, because these bacteria can withstand endodontic procedures and infiltrate the tiny channels within the tooth's structure known as dentinal tubules [44, 45]. To treat or prevent endodontic lesions, standard endodontic therapy focuses on lowering the microbial load within the root canal system. To better debride and cleanse the root canal system, various endodontic procedures employ chemo-mechanical treatment plus irrigation with disinfectants [46]. The most common solution used in endodontic treatment is sodium hypochlorite (NaOCl) because of its well-established efficacy against bacteria and other pathogens [47]. However, ecchymosis, tissue necrosis, and paresthesia are some of the unintended consequences of NaOCl use [48]. It has therefore been proposed that, rather than resorting to systemic antibiotics, it would be desirable to design a locally administered therapeutic agent with antibacterial activity that can reach into the dentinal tubules. Root canal bacteria could in theory be reduced or eliminated using laser technology in a way that is both noninvasive and effective [49, 50].

Visible light irradiation can be used to inactivate bacteria without the use of any chemical compounds, solvents, or other additives. Several different types of bacteria and fungi have been disclosed to be sensitive to this visible light disinfection technique. The use of light alone to kill microorganisms is known as antimicrobial photoinactivation (aPI) [51]. On the other hand, in antimicrobial photodynamic therapy (aPDT) a photosensitizer must be added from outside to allow the chemical reaction between light and oxygen to take place. Oxidative damage to the bacteria is caused by the ROS produced during this process [52]. aPI is advantageous since it kills bacteria without harming the host [51]. It has been observed that the usage of aPI within the visible light range results in rapid, effective, and locally targeted antibacterial action [23, 53], with little or no collateral impairment to the surrounding host tissue [23]. Consequently, the use of laser-based aPI for a variety of medicinal purposes is now being considered. Additionally, given the multi-target properties inherent in ROS, the probability of inducing bacterial resistance through aPI is lessened in comparison to antibiotics [54–56]. Compared to high-powered lasers, the cost of aPI is far lower, and the procedure itself is rapid and painless [57].

Few studies have shown that lasers were capable of eliminating *E. faecalis* completely [58, 59]. The efficacy of aPI is reliant on many aspects, comprising the spectral range of the light source and the ability of the PS inside the bacteria to absorb the radiation [60]. To maximize the effectiveness of aPI, optimizing the laser wavelength is critical.

In this study, vancomycin-resistant *E. faecalis* V583 was irradiated with femtosecond laser light tuned to different wavelengths between 420 and 465 nm initially at a fixed fluence of 1000 J/cm^2^ to determine the most effective wavelength to inactivate the bacteria. We employed VRE in this study, as a worst-case scenario, because the American Society for Microbiology (ASM) recommends using a strain that is exceptionally resistant compared to most bacteria when testing disinfection strategies such as femtosecond laser therapy [61]. To our knowledge, there are no studies available reporting the inactivation of VRE with visible light in the range of 420 – 465 nm. The irradiated bacteria were harvested from a stationary growth phase of an overnight culture because endogenous bacterial PS such as porphyrins or flavins are only produced at certain stages of the growth cycle (lag, exponential, or stationary), making the value of the aPI effect conditional on the stage of bacterial growth [10, 62].

**Fig. 2:**
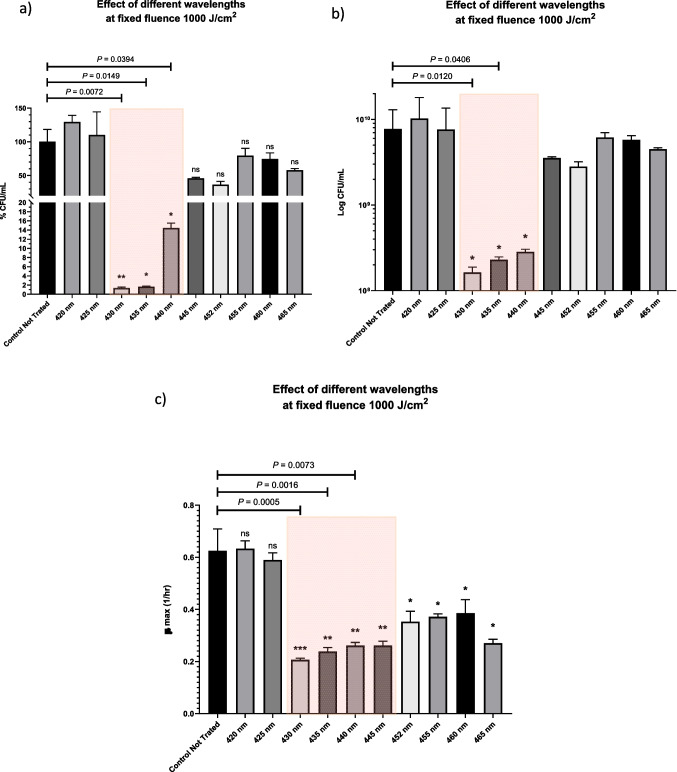
The bar graph compares the control culture to bacterial cultures that were irradiated with different Laser parameters at ten specific wavelengths: 420, 425, 430, 435, 440, 445, 452, 455, 460, and 465 nm at a fixed fluence of 1000 J/cm^2^ in terms of (a) percentage CFU/mL, (b) log CFU/mL, and (c) the growth rate (μ_max_) during the logarithmic phase of growth. Statistical significance was determined through ANOVA followed by the Tukey test, with the level of significance indicated by asterisks: *** denotes highly significant (*P* < 0.001), ** denotes moderately significant (*P* < 0.01), * denotes low significance (*P* < 0.05), and "ns" represents not statistically significant

Colony-forming units (CFU) were utilized to enumerate viable bacterial abundance in each sample, and the results were statistically compared. The viable bacterial count (CFU/mL) and the percentage of viable bacterial cells before and after laser irradiation at different wavelengths were plotted in (Figs. 2A and 2B). The highest effective wavelengths were 430 nm and 435 nm, causing nearly 2 log reduction (98.6% and 98.3% inhibition, respectively) of viable bacteria counts, and this is close to the ASM recommendation that a 99% reduction is needed in bactericidal testing [63]. Other researchers have adopted a 90% reduction in CFUs as a target value to be achieved using bactericidal radiation [33, 64–66].

440 nm caused 85.6% decrease in viable bacteria counts. Other wavelengths caused a decrease in the viable bacterial counts, but these were not statistically significant. The percentage of killed bacteria treated with 445, 452, 455, 460 and 465 nm were 54.2%, 63.67%, 20.5%, 25.5% and 42%, respectively. Both 420 nm and 425 nm showed no bactericidal effects against *E. faecalis*.

Our findings are consistent with those of other groups who showed enhanced disinfection but did not observe complete eradication of bacteria (100%). Therefore, femtosecond lasers could be employed in conjunction with other disinfectant agents [67]. In addition to irrigation solutions, the use of femtosecond lasers may accelerate the rate at which bacteria are eliminated [68, 69]. Previously, it was reported that a high-power laser eliminated only 97% of *E. faecalis* [6, 70]. Castelo et al. [71] employed a 940 nm diode-laser to kill *E. faecalis*, and found that 70% of the bacteria were eradicated. With an 810 nm diode laser, Gutknecht et al. [59] found that 74% of bacterial colonies were destroyed. Some diode lasers have been displayed to be > 95% effective towards bacteria, according to other studies [58, 70].

In this study, a higher energy density of 1000 J/cm^2^ was required to achieve 98.6% killing of *E. faecalis*. This is consistent with other reports which showed that visible light was more effective against *S. aureus*, *A. baumannii,* and *P. aeruginosa* than it was against *Enterococci*, *E. coli,* or *K. pneumoniae*, which required higher doses to achieve just a 1 log reduction [72]. Using *E. faecium* isolated from clinical sample, Halstead et al. [33] reported that 393 J/cm^2^ of 400 nm irradiation was required for a 1 log reduction, while Hones et al. [72] reported that 525 J/cm^2^ was needed for the same result. To disinfect drinking water against *E. faecalis*, Lui et al. [66] reviewed the effects of 455 nm irradiation and reported that an exposure level of 410 J/cm^2^ was necessary for a 1 log reduction.

Moreover, growth curves (Fig. S1) and maximum growth kinetics (μ_max_) of the bacteria at specific time points (Fig. 2C) were plotted before and after laser irradiation to monitor the differences in growth rates. The growth rate kinetics were significantly different after femtosecond laser treatment with wavelengths from 430 to 465 nm compared to the control untreated bacteria. It is worth mentioning that by slowing the growth rate kinetics, treatment with other antibacterial agents would be made more effective and synergistic effects could be observed. In the growth curves of the femtosecond laser treated bacteria with wavelengths between 430 and 465 nm, there was an increase in the lag phase, and the maximum growth was obtained after a longer time compared to 6 h in the case of control bacteria.

According to the findings of this study, the wavelengths that exhibited the greatest effectiveness were 430 and 435 nm. Bacterial cells contain endogenous photosensitizers that are responsible for their inactivation when exposed to visible light. These photosensitizers are excited when they absorb photons of the correct wavelength. ROS can be generated when the excited state PS undergoes a reaction with atmospheric oxygen in its ground state [11], which can then oxidize multiple intracellular targets at a molecular level [73–78].

Evidence supporting the importance of ROS comes from studies in which the cellular photodamage effect was partially blocked by pre-treating a suspension of microorganism cells with a recognized ROS scavenger before irradiation with a laser [36, 42, 79]. Indeed, blue light can cause damage to microorganisms in a number of ways, including the disruption of membrane integrity [34], the oxidation and release of DNA [75, 80], low levels of ATP inside the cell as a result of ATPase inhibition [75], harmful effects on vital metabolic enzymes [75], and disturbances to a number of metabolites such as coformycin, actinonin, 11-deoxycortisol, chitobiose, and tyramine [81]. Exposure to blue light has a photochemical effect rather than a photothermal effect. In fact, when microorganisms in a suspension had their temperature rise tracked using a thermocouple during irradiation, it did not surpass 0.5–4.0 °C, and the median temperature was below the point at which microorganisms are thought to die [39, 64].

Endogenous photosensitizer composition and concentration are the main factors governing bacterial damage after light irradiation. The role of porphyrin or flavin photosensitizers in the growth inhibition of microorganisms produced by blue light radiation is pathogen-specific [43]. The contribution of porphyrin photosensitizers is maximized by radiation with a wavelength of 405 nm (the Soret band absorption maximum), while the contribution of flavins is greatest when exposed to radiation at 430—450 nm, particularly 445 nm, which is the flavin absorption maxima and is barely noticeable in porphyrin absorption spectra [82]. For clinical applications, the blue wavelength of 450 nm may be preferable to 405 nm because of its deeper tissue penetration and reduced absorption by blood [25, 32, 43]. Flavin fluorescence is easily detected in extracts of *S. aureus* and *Enterococcus* s*pp*., while porphyrin fluorescence is barely detectable [43].

Most reports have focused on targeting porphyrins in the range of 390–420 nm. But *E. faecalis* is an exception to this rule due to no endogenous porphyrin content but does have flavins as the main photosensitizers. *E. faecalis* cannot synthesize its own porphyrins, but instead must rely on taking up heme from its environment as a source of the porphyrins needed for enzyme co-factors [83]. Maximum fluorescence emission from flavin compounds occurs between 520 and 525 nm, while the excitation spectrum for fluorescence has two peaks, at 370 and 435 nm [43]. This is consistent with the results of our study, at which the maximum antimicrobial activity was noticed between 430 and 435 nm, indicating that the efficacy was due to absorption by flavins rather than porphyrins. Experimental evidence has shown that compounds of the flavin class could act as absorbers of blue spectrum light, followed by sensitizing the generation of reactive oxygen species [43, 72].

**Fig. 3:**
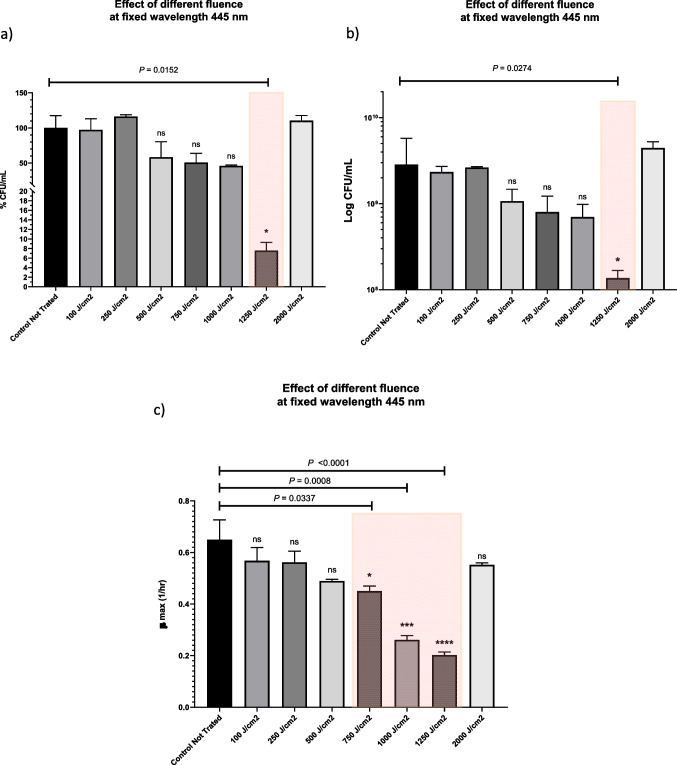
The bar graph compares the control culture to bacterial cultures that were irradiated fixed laser wavelength of 445 nm at different fluences of 100, 250, 500, 750, 1000, 1250 and 2000 J/cm^2^ in terms of (a) percentage CFU/mL, (b) log CFU/mL, and (c) the growth rate (μ_max_) during the logarithmic phase of growth. Statistical significance was determined through ANOVA followed by the Tukey test, with the level of significance indicated by asterisks: **** denotes the highest significance (*P* < 0.001), *** denotes highly significant (*P* < 0.001), * denotes low significance (*P* < 0.05), and "ns" represents not statistically significant

Although our preliminary results showed that the maximum antibacterial activity was at 430 and 435 nm, we also investigated the wavelength of 445 nm, which was previously reported to be a maximum in the flavin absorption spectrum. Our investigation evaluated the antibacterial activity of femtosecond laser at 445 nm with different fluences of 100, 250, 500, 750, 1000, 1250, and 2000 J/cm^2^. Then the viable bacterial count (CFU/mL) and the percentage of viable bacterial cells before and after laser irradiation were plotted in (Fig. 3A and Fig. 3B). Moreover, growth curves (Fig. S2) and maximum growth kinetics (μ_max_) of the bacteria at specific time points (Fig. 3C) were graphed before and after laser irradiation to monitor the growth rate differences at each tested fluence. Physically, the term fluence or energy density refers to the amount of energy imparted by electromagnetic radiation per unit area. Fluence equals (*P*_*ave*_* /* $\pi$ r^2^) $\times T$, where the average laser power is denoted by *P*_*ave*_, the laser beam radius is denoted by r, and the exposure duration is denoted by T. This means that an increase in either the average power or the exposure time will result in an increase in energy density.

A progressive decrease in colony-forming units was observed after a corresponding increase in energy density at a fixed wavelength of 445 nm, and the most effective fluence was 1250 J/cm^2^ causing 92.5% inhibition (more than 1 log) of viable bacteria counts. Also, the growth rate kinetics were progressively altered after femtosecond-laser treatment at 445 nm using a gradual increase in energy density. In particular, at an energy density of 1250 J/cm^2^, laser-exposed cultures grew substantially more slowly than control cultures.

Our results are consistent with those of previous studies indicating that the inactivation rate of many bacteria increases with increasing doses of irradiation at different wavelengths: 400 nm [33], 405 nm [34, 36, 42, 80], 460 nm [65] for *E. coli*; 405 nm [36] and 415 nm [84] for *C. albicans*; 400 nm [33], 405 nm [64, 80], 520 nm [64] and 400–550 nm [40] for *S. aureus*. This behavior may have a rational explanation: a higher number of photons will increase the probability of them interacting with photosensitizers and inducing more ROS production; hence, increasing the antibacterial photoinactivation effect [15, 85].

**Fig. 4:**
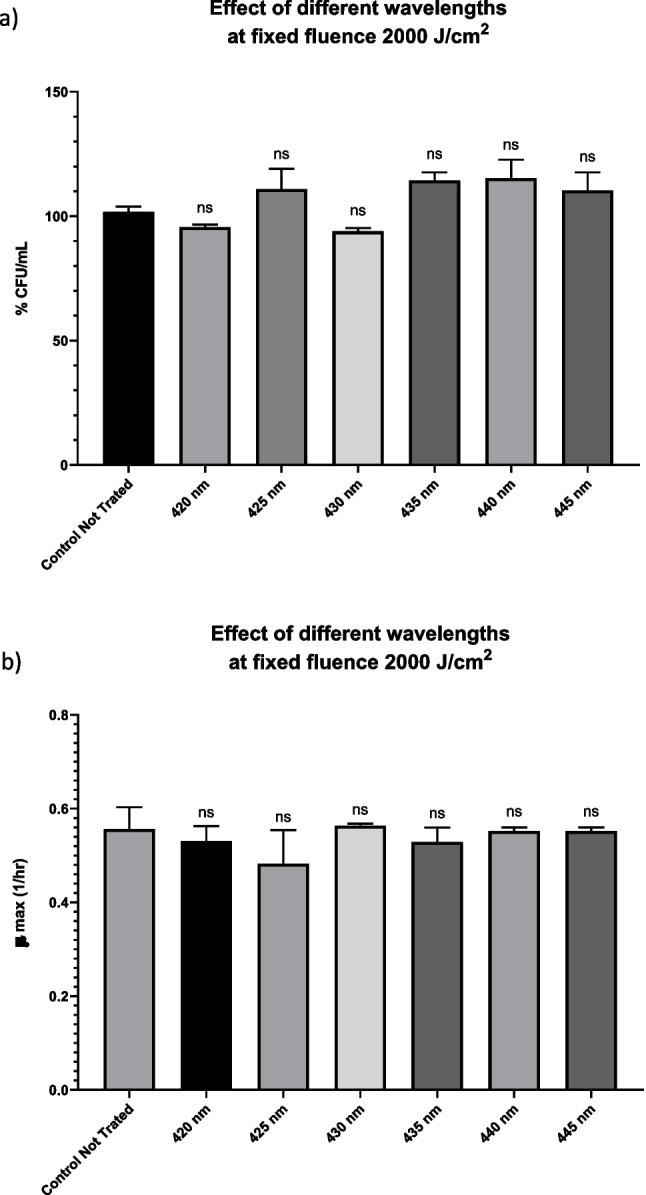
The bar graph compares the control culture to bacterial cultures that were irradiated with different Laser parameters at ten specific wavelengths: 420, 425, 430, 435, 440, 445, 452, 455, 460, and 465 nm at a fixed fluence of 2000 J/cm.^2^ in terms of a) percentage CFU/mL, and c) the growth rate (μ_max_) during the logarithmic phase of growth. (ns; not-significant)

Previously, it was reported that Enterococci required higher irradiation doses for effective photoinactivation compared to other species, and microbial cell photodestruction increases with the energy dose up to a relatively high-level [42]. For this reason, we doubled the irradiation dose to 2000 J/cm^2^ using wavelengths from 420 to 445 nm in order to investigate the antimicrobial efficacy. The percentage of viable bacterial cells before and after laser irradiation were plotted in Fig. 4A. Moreover, growth curves (Fig. S3) and maximum growth kinetics (μ_max_) of the bacteria at specific time points (Fig. 4B) were plotted before and after laser irradiation.

Surprisingly, after applying a fluence of 2000 J/cm^2^, the efficacy was reduced and we got less inhibitory effects in terms of viable bacterial counts. This observation could be attributed to a photobleaching phenomenon of the flavin molecules arising at a high energy density of 2000 J/cm2. Also, the growth rate kinetics were not altered compared to the control bacteria for all laser treatment wavelengths. The findings here are in line with those of other studies that reported higher energy doses of radiation were required to detect a pronounced bactericidal effect [32, 39], but photobleaching phenomena could be considered at certain high energy levels. Contrary to the findings of some earlier studies [86–88], we found that raising the energy density beyond a specific value had no additional effect on reducing the bacterial viability. A reasonable explanation as mentioned, could be the occurrence of the phenomenon known as photobleaching, an irreversible process in which the excitation light triggers chemical reactions that could destroy the molecular structure of a particular photosensitizer [89]. The temperature rise produced, and the excitation light power both influence the photobleaching process. Therefore, the photosensitizer molecule is damaged when the energy density is increased beyond a specific threshold, which then affects ROS production and, in turn, reduces the bactericidal action [86, 90, 91]. This phenomenon highlights the need to optimize not only the wavelength, but also the energy density for most effective aPI implementation.

## Conclusions

Antibacterial photoinactivation treatment might be a revolutionary, non-invasive therapy for Vancomycin-resistant *Enterococcus faecalis.* As far as we are aware, this is the first study to report the deactivation of VRE with visible light. The most effective wavelengths were 430 and 435 nm at a fluence of 1000 J/cm^2^, causing nearly 2 log reduction (98.6% and 98.3% inhibition, respectively) of viable bacteria counts, suggesting the participation of intracellular flavins rather than intracellular porphyrins in the photodynamic bactericidal effect. Bacterial survival and growth kinetics are clearly affected by an increase in energy density, but only up to a certain point. The colony-forming units and growth rate of the laser-treated cultures were progressively decreased as energy density or light dose increased at a fixed wavelength of 445 nm, at which the most effective fluence was 1250 J/cm^2^ causing 92.5% inhibition (more than 1 log inhibition) of viable bacteria counts. Surprisingly, after applying a fluence of 2000 J/cm^2^, the efficacy was reversed and less inhibitory effects in terms of viable bacterial counts were recorded. This could be attributed to photobleaching of the flavins. Our results highlight the importance of optimising laser exposure parameters such as wavelength and energy density and their effect on improving the efficacy of aPI.

## Supplementary Information

Below is the link to the electronic supplementary material.

Supplementary file1 (DOCX 304 KB)

## Data Availability

Data will be made available on request.
