# Supplementary material for: Studying the viability and growth kinetics of vancomycin-resistant Enterococcus faecalis V583 following femtosecond laser irradiation (420–465 nm)
Source: Lasers Med Sci. 2024 May 29;39(1):144. doi: 10.1007/s10103-024-04080-5 (PMC11136855; doi:10.1007/s10103-024-04080-5)
Supplement: Supplementary file 1 — Supplementary file1 (DOCX 304 KB) [file 10103_2024_4080_MOESM1_ESM.docx]

**Studying the viability and growth kinetics of vancomycin-resistant Enterococcus faecalis V583 following femtosecond laser irradiation (420–465 nm)**

**Figure S1**: Growth curves of femtosecond laser-treated bacteria relative to control untreated bacteria at a fixed fluence of 1000 J/cm^2^ with different wavelengths; a) 420 nm, b) 425 nm, c) 430 nm, d) 435 nm, e) 440 nm, f) 445 nm, g) 452 nm, h) 455 nm, i) 460 nm, and j) 465 nm.

**Figure S2**: Growth curves of femtosecond laser-treated bacteria relative to control untreated bacteria at a fixed wavelength of 445 nm with different fluences; a) 100 J/cm^2^, b) 250 J/cm^2^, c) 500 J/cm^2^, d) 750 J/cm^2^, e) 1250 J/cm^2^, and f) 2000 J/cm^2^

**Figure S3**: Growth curves of femtosecond laser-treated bacteria relative to control untreated bacteria at a fixed fluence of 2000 J/cm^2^ with different wavelengths; a) 420 nm, b) 425 nm, c) 430 nm, d) 435 nm, e) 440 nm, and f) 445 nm.
